# Comparison of short-time functional outcomes after TKA between Multigen Plus CR and Physica KR prostheses

**DOI:** 10.1007/s12306-021-00730-x

**Published:** 2021-10-19

**Authors:** P. Lisý, J. Čabala, M. Hrubina, M. Melišík, L. Nečas

**Affiliations:** 1 Orthopaedic Clinic of University Hospital Martin, Kollárova 2, Martin, 036 59 Slovak Republic; 2 Jessenius Faculty of Medicine in Martin, Comenius University in Bratislava, Malá Hora 10701/4A, Martin, 036 01 Slovak Republic

**Keywords:** Total knee arthroplasty, Physica KR, Multigen plus CR, Functional outcomes, Kinematics-retaining

## Abstract

**Background:**

Aim of this study was to compare functional results within 36 months following primary total knee arthroplasty (TKA) using a conventional prosthesis Multigen Plus CR and a new Physica KR implant. Our hypothesis was that the use of the Kinematics-Retaining design of an TKA implant leads to a significantly greater improvement in the active range of motion and better functional results (KSS 1, KSS 2 and WOMAC score) than the conventional CR implant at short-term follow-up.

**Materials and methods:**

We retrospectively analysed data of 234 patients who underwent primary TKA at our hospital from April 2010 to August 2015 with the CR type of implant and from July 2014 to August 2015 with the KR implant due to advanced knee arthrosis of IIIrd and IVth grade of Kellgren-Lawrence classification, with no major ligamentous instability. Western Ontario McMaster Universities Osteoarthritis Index (WOMAC) questionnaire, Knee Society Scores 1 (KSS 1) and 2 (KSS 2) and flexion (AROM) were recorded preoperatively and at 6, 12 and 36 months after surgery.

**Results:**

Our study showed a statistically significant difference in functional results at three years with better KSS 1 score, a tendency to higher values in the KSS 2 score, as well as a statistically significant overall improvement in AROM in favour of the new KR design over the conventional CR implant with a post-hoc power analysis of 83.8%. We found that there was no statistically significant difference between groups when comparing WOMAC score and complications at short-term follow-up.

**Conclusions:**

Our study provided more favourable clinical results for using Kinematics-Retaining implant in primary TKA. Further studies should focus on radiological and functional outcomes from mid- to long-term follow-up.

## Introduction

Despite the positive long-time survivorship of conventional total knee arthroplasty (TKA), a significant percentage of patients remain dissatisfied after surgery, with authors reporting patients being unsatisfied from 15 to 20% [1, 2], and from 30 to 50% [3, 4]. This is most often due to residual pain as well as to reduced mobility relating to serious limitations in activities of daily living [2, 5–7]. According to the Slovak Arthroplasty Registry, the overall revision rate after primary TKA is 4.33% [8]. Nonetheless, this information does not provide us any indication regarding the level of patient satisfaction. It has been known for a long time that the satisfaction rate after the primary TKA is significantly lower than after the primary total hip replacement [9]. Nowadays, because life expectancy has been increasing, the number and age spectrum of patients undergoing TKA has increased significantly. It is therefore necessary to adapt implants and their use to specific individual patient requirements, although some limitations may remain unchanged.

It has been proved that endoprosthesis design has a direct impact on postoperative outcome and patient satisfaction [10], affecting directly the kinematics of the knee joint, and thus stability during movement [11]. In this study, we compared two designs of primary TKA, Multigen Plus CR (Limacorporate, Italy) and Physica KR (Limacorporate, Italy). Multigen Plus CR has been using since 1997, thus for more than 20 years, and it can be considered as a conventional and reliable endoprosthesis. It is a cemented TKA that retains the posterior cruciate ligament and provides five options of size matching for each component size, but it only presents six component sizes overall, with the smallest tibial component which can be used only with the smallest femoral component and vice versa. On the other hand, Physica KR (Kinematics-Retaining) is a new type of implant that was launched in 2015. As the Multigen Plus CR, it is a cemented and PCL retaining knee. Its difference consists in the shape of the KR femoral condyles in the sagittal plane which shows multiple radii of curvature. The J-curve allows the femur to physiologically stretch the collateral ligaments throughout the flexion–extension cycle. Moreover, the Physica KR presents an asymmetric shape of the tibial insert with a medial concave shape, and a lateral convex or “saddled” shape that reproduces the natural roll-back movement and femoral rotation through the tibia, thereby reducing sliding friction. The Physica KR offers ten femoral and ten tibial component sizes, each of them is compatible with ± 2 sizes of the other component, respectively. A total of five options for each tibial or femoral component size are available.

Our hypothesis was that the use of the Kinematics-Retaining design of an TKA implant leads to a significantly greater improvement in the active range of motion and better functional results (KSS 1, KSS 2 and WOMAC score) than the conventional CR implant at short-term follow-up. The aim of the study was to compare functional results after primary TKA between these two designs within 36 months after surgery.

## Materials and methods

We retrospectively analysed data of 234 patients who underwent primary TKA at our hospital from April 2010 to August 2015 with the conventional CR TKA implant and from July 2014 to August 2015 with the new KR TKA implant. Most patients were indicated for TKA due to advanced knee arthrosis IIIrd and IVth grade of Kellgren-Lawrence classification [12], with no major ligamentous instability, thus retaining their posterior cruciate ligament. Ethics Committee (EK UNM č. 174/2019, 27/11/2019) granted approval to proceed in this study.

All operations were performed by four experienced surgeons (> 150 cases per year) according to standardised surgical procedure, which was used both in the CR and KR groups. In all cases, a mid-vastus surgical approach was used without patellar replacement. Only reshaping of patella was performed, without denervation; the patella was lateralised without eversion during the operation. In all cases, a ligament balancer (tensiometer) was used to balance soft tissues in extension and at 90° flexion as well, thus, to determine the rotation of the femoral component [13]. First generation of cephalosporins were administered, clindamycin was used in penicillin-allergic patients. Prevention of thromboembolism was performed according to current standard recommendations.

The same standardised post-operative rehabilitation protocol was used for every patient. It included: early mobilisation, which was performed already on the first postoperative day, toning exercises, exercises on continuous passive motion knee device and walking with the crutches under the supervision of a physiotherapist. The following days rehabilitation program included active exercises, walking with crutches on the flat floor and finally walking up and down the stairs. After having mastered the standardised rehabilitation procedures, patients were discharged from hospital to outpatient care on the fourth to tenth postoperative day (i.e. on the sixth day, averagely).

Functional results were evaluated using the Western Ontario McMaster Universities Osteoarthritis Index (WOMAC) questionnaire [14], the Knee Society Score 1 (KSS 1) and Knee Society Score 2 (KSS 2), as well as the active range of motion—flexion (AROM), which was measured with a goniometer. Active flexion was recorded using the SFTR method. We performed the assessments preoperatively and at 6, 12 and 36 months after surgery. All patients who completed the follow-up control at 36 months till end of December 2018 were included in the study; patients who could not complete this control due to revision of the knee joint were also recorded in this study.

The obtained data were processed in the statistical program Real Statistics (Microsoft Excel 2016), with the significance level at *p* < 0.05. The Shapiro-Wilcoxon test was used to determine the Gaussian distribution of values. When comparing values between groups the Student's *t*-test was used for normal distribution of values (BMI, age) and Mann–Whitney *U* test for nonparametric values (others). The Wilcoxon Signed-Rank test was used to evaluate the results within each group.

We also recorded all implant-related complications within 3 years after surgery. X-ray analysis and evaluation of non-specific complications [15, 16] were not part of this study.

## Results

**Figs. 1 and 2:**
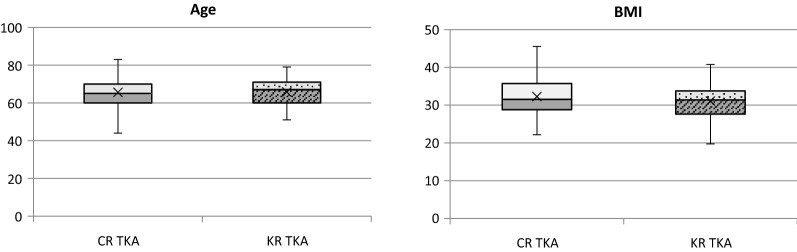
Age and BMI distribution in KR TKA and CR TKA groups

We analysed a total of 234 primary TKAs, 162 in the CR TKA group and 72 in the KR TKA group. 41 patients in the CR group and 22 in the KR group underwent one-time bilateral knee replacement. Most patients in both groups were indicated for advanced primary knee arthrosis (CR: 98.1%, KR: 98.6%). Comparison of demographic data is shown in Table 1, Figs. 1, 2. In the CR group, the male to female ratio was approximately 1: 2, while it resulted 1: 3 in the KR group (*p* = 0.06). The mean age was 65.6 ± 7.4 years in the CR group and 65.8 ± 6.9 in the KR group, without any significant difference (*p* = 0.77). The mean BMI was 32.3 ± 4.6 in the CR group and 31.1 ± 4.7 in the KR group, which resulted as statistically nonsignificant (*p* = 0.08). The homogeneity of the two groups for age, BMI and gender was then confirmed, although there was a higher percentage of male patients and a higher average BMI in the CR group.

**Table 1:** Demographic data in KR TKA and CR TKA groups

	CR TKA (*n* = 162)	KR TKA (*n* = 72)	*p-*value
Gender M/ F	56/106	16/56	0.06
Unilateral/ bilateral TKA	80/41	28/22	
Primary osteoarthritis grade III–IV /rheumatoid/ post-traumatic	159/2/1	71/1/0	
Age (mean ± SD)	65.6 ± 7.4	65.8 ± 6.9	0.77*
BMI (mean ± SD)	32.3 ± 4.6	31.1 ± 4.7	0.08*

*Student’s *t*-test

**Fig. 3:**
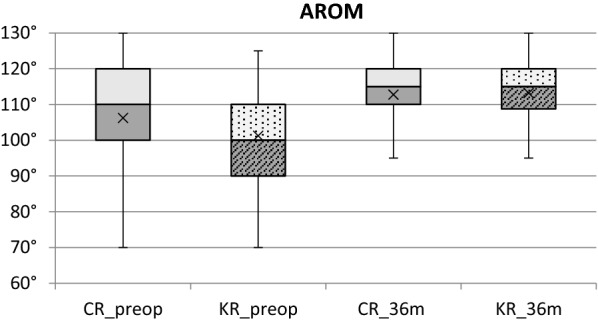
Overview of active range of motion (AROM) preoperatively and at 36 months after surgery for KR TKA and CR TKA groups

In the AROM assessment (Table 2, Fig. 3), it was reported a statistically significant difference with better flexion in the CR group with respect to the KR group preoperatively, with 106.2^◦^ ± 14.5^◦^ and 101.3^◦^ ± 14.2^◦^ (*p* < 0.05), respectively. Postoperatively, both in the CR and KR groups, there was a statistically significant improvement at follow-up. At 6 and 12 months, there was no significant difference in AROM between the two groups, but the overall improvement at 36 months was more significant in the KR than in CR group (*p* < 0.005) with post-hoc power analysis at 83.8%.

**Table 2:** Mean Active range of motion (AROM)—flexion in KR TKA and CR TKA groups

AROM		CR TKA (*n* = 162)		KR TKA (*n* = 72)		*p-*value (Mann–Whitney *U* test)
	preop	106.2° ± 14.5°		101.3° ± 14.2°		0.008
			*p* < 0.005*		*p* < 0.005*	
	6 m	108.4° ± 12.8°		106.5° ± 10.9°		0.09
			*p* < 0.005*		*p* < 0.005*	
	12 m	111.3° ± 12.5°		110.3° ± 11.0°		0.34
			*p* < 0.005*		*p* < 0.005*	
	36 m	112.8° ± 12.2°		113.2° ± 10.6°		0.99
δ improvement ± SD		6.5° ± 12.8		11.9° ± 13.0°		0.004

*Wilcoxon Signed-Rank test—comparison within the group in each follow-up interval)

Post-hoc power analysis: 83.8%

**Fig. 4:**
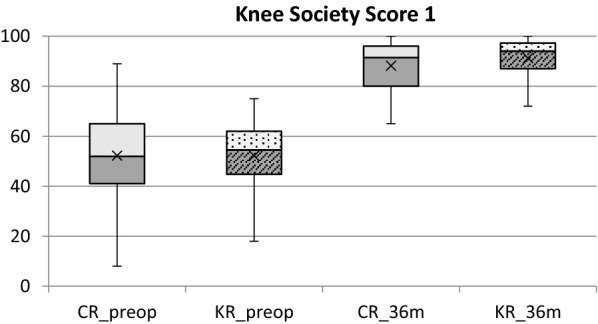
Overview of KSS 1 score preoperatively and at 36 months after surgery for KR TKA and CR TKA group

Knee Society Score (KSS) consists of two parts, KSS 1 and KSS 2, each ranging from 0 to 100 points (with 100 being the maximum score). KSS 1 evaluates the knee itself (i.e. knee pain during movement, active range of movement—flexion, deficit of extension, lateral stability, anterior–posterior stability and axial deformity of the limb). When evaluating KSS 1 score (Table 3, Fig. 4), we did not find a statistically significant difference preoperatively (*p* = 0.93) between the two groups. At 6 and 12 months, we did not observe a statistically significant difference in KSS 1, whereas at 36 months the KR group score was significantly better with respect to the CR group, 91.3 ± 9.9 and 88.2 ± 9.3 (*p* < 0.05), respectively, with post-hoc power analysis at 73,9%. When evaluating the KSS 1 score within each group, we had a statistically significant improvement in both CR and KR group at 6 and 12 months (*p* < 0.005). After 12 months, there was no significant improvement in KSS 1 score in either group (*p* = 0.29 and *p* = 0.14, respectively).

**Table 3:** Comparison of Mean Knee Society Score part 1 between KR TKA and CR TKA TKA group

KSS 1		CR TKA (*n* = 162)		KR TKA (*n* = 72)		*p-*value
	Preop	52.3 ± 17.5		52.2 ± 12.0		0.93
			*p* < 0.005*		*p* < 0.005*	
	6 m	86.9 ± 9.6		87.5 ± 9.0		0.69
			*p* < 0.005*		*p* < 0.05*	
	12 m	89.1 ± 8.5		89.9 ± 7.8		0.63
			*p* = 0.29*		*p* = 0,14*	
	36 m	88.2 ± 9.3		91.3 ± 8.0		0.03

*Wilcoxon Signed-Rank test

Post-hoc power analysis: 73.9%

**Fig. 5:**
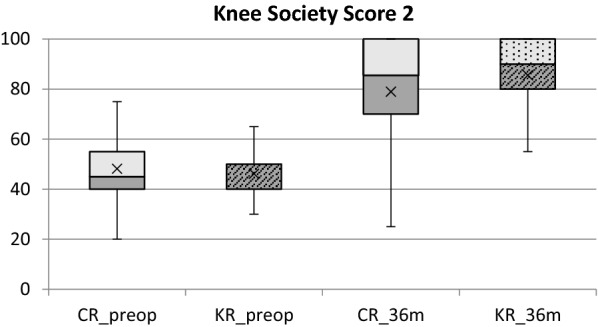
Overview of KSS 2 score preoperatively and at 36 months after surgery for KR TKA and CR TKA group

The KSS 2 score evaluates the patient's functionality (i.e. use of crutches, walking stairs and walked distance). The KSS 2 score (Table 4, Fig. 5) did not show as well any statistically significant difference preoperatively (*p* = 0.84). At 6, 12 and 36 months, we did not observe a statistically significant difference in KSS 2 scores between the two groups, although at 36 months there was a tendency for higher values in the KR group with respect to the CR group, 85.5 ± 20.4 and 78.9 ± 24.9 (*p* = 0.08), respectively, with post-hoc power analysis at 56,7%. When evaluating the KSS 2 scores within the groups, we had a significant improvement in both CR and KR group (*p* < 0.005) at 6 and 12 months. At 12 months, there was no significant improvement in KSS 2 scores in either group, although in the KR group there was a trend toward higher values (*p* = 0.37 and *p* = 0.09, respectively).

**Table 4:** Comparison of Mean Knee Society Score part 2 between KR TKA and CR TKA TKA group

KSS 2		CR TKA (*n* = 162)		KR TKA (*n* = 72)		*p*-value
	Preop	48.1 ± 13.2		46.1 ± 14.1		0.84
			*p* < 0.005*		*p* < 0.005*	
	6 m	77.6 ± 16.9		76.8 ± 17.5		0.72
			*p* < 0.005*		*p* < 0.005*	
	12 m	82.6 ± 18.6		84.1 ± 17.5		0.74
			*p* = 0.37*		*p* = 0.09*	
	36 m	78.9 ± 24.9		85.5 ± 20.4		0.08

*Wilcoxon Signed-Rank test

Post-hoc power analysis: 56.7%

**Fig. 6:**
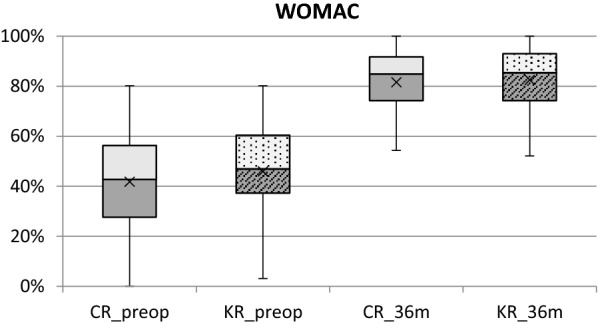
Overview of WOMAC score preoperatively and at 36 months after surgery for KR TKA and CR TKA group

WOMAC scores reflect the patient's subjective evaluation. There was no statistically significant difference in WOMAC neither preoperatively (*p* = 0.14), nor at 6, 12 and 36 months postoperatively (*p* = 0.39, 0.53, 0.71, respectively) between the two groups (Table 5, Fig. 6). Within each group, there was a very similar trend in terms of increasing WOMAC scores at 6 and 12 months, showing a statistically significant improvement at these follow-ups (*p* < 0.005). However, there was no further significant improvement in WOMAC after 12 months in both groups (*p* = 0.10 and *p* = 0.28, respectively).

**Table 5:** Comparison of Mean Western Ontario McMaster Universities Osteoarthritis Index (WOMAC) between KR TKA and CR TKA group

WOMAC		CR TKA (*n* = 162)		KR TKA (*n* = 72)		*p*-value
	Preop	41.8% ± 19.2		45.9% ± 17.7		0.14
			*p* < 0.005*		*p* < 0.005*	
	6 m	77.3% ± 12.8		78.7% ± 13.2		0.39
			*p* < 0.005*		*p* < 0.005*	
	12 m	80.6% ± 12.6		81.8% ± 12.8		0.53
			*p* = 0.10*		*p* = 0.28*	
	36 m	81.6% ± 14.6		82.6% ± 13.4		0.71

*Wilcoxon Signed-Rank test

Post-hoc power analysis: 7.4%

The most frequent complication in both groups was persistent mild pain (Table 6): 48 (29.6%) cases in the CR group and 18 (25%) cases in the KR group (*p* = 0.57). Stiffness was 6.7% in the first group and 9.7% in the second group (*p* = 0.72). One revision due to early periprosthetic infection in the CR group was performed with good results. Neither periprosthetic infection nor aseptic loosening were observed in KR group. No case of perioperative complications and deep venous thrombosis were observed. Moreover, no cases of very rare complications were recorded [17].

**Table 6:** Comparison of the incidence of Complications between KR TKA and CR TKA group

	CR TKA (*n* = 162)	KR TKA (*n* = 72)	*p*-value
Residual mild pain	48/29.6%	18/25%	0.57*
Stiffness	11/6.7%	7/9.7%	0.72*
Surgical site infection	1	0	
Deep vein thrombosis	0	0	
Revision/ loosening TKA	1	0	

*Mann–Whitney *U* test)

## Discussion

The aim of this study was to compare functional results after primary TKA at three-year follow-up between a conventional CR type of endoprosthesis and a new kinematics-preserving design of an implant. At the beginning of the study, it was assumed that this new design of an TKA implant leads to a significantly greater improvement in the active range of motion and better functional results (KSS 1, KSS 2 and WOMAC score) than the conventional CR implant at short-term follow-up. Our study showed a statistically significant difference in functional results with better KSS 1 scores at three years, a tendency to higher KSS 2 score, as well as a statistically significant overall improvement in active range of movement in favour of the KR group versus the CR group, thus confirming our study hypothesis.

 On the other hand, there was no statistically significant difference in WOMAC scores between the two groups at short-term follow-up. We have also observed very good functional results both in CR and in the KR group, with almost 100% survivorship at three years aside for one revision due to infection in the CR group.

Although there were no statistical differences in AROM postoperatively between the two groups, the overall improvement in flexion was significantly better in the KR group at 36 months. Thus, we indirectly disproved the findings of Shi et al. [18] which indicated that patients with worse functional outcomes and poor mobility prior to surgery are worse even after surgery.

By statistical analysis of the KSS 1 and KSS 2 scores in the two groups, we found an almost identical trend, with no statistically significant difference between the CR and KR group preoperatively (Tables 3 and 4). Both the KR and the CR groups showed a statistically significant improvement with respect to preoperative values at 6 and 12 months. At 36 months, KSS 1 scores indicated a statistically significant increase in the KR group with respect to the CR group, and the KSS 2 showed a tendency to higher values as well in the KR group, which may be of greater importance to patients in the long term.

As the overall WOMAC score reflects the subjective evaluation of patients, according to Walker et al. [19] it can be used as a reliable method for the assessment of patient satisfaction. In this study, there were no statistically significant differences between CR and KR groups at follow-up in terms of WOMAC outcomes (Table 5). A similar trend was noted in terms of improvement at follow-up, with patients of both groups already experiencing a significant increase in WOMAC at 6 and 12 months (*p* < 0.005); at 36-month follow-up, patients in both groups did not show any additional significant improvement in their WOMAC scores (*p* = 0.10; *p* = 0.28, respectively). Walker et al. [19] suggested that the WOMAC classification derives according to the patient satisfaction based on a retrospective study conducted by the National Joint Registry in the UK. This study compared WOMAC scores, SF-12 scores and satisfaction questionnaires in 2589 patients after primary TKA. Based on the comparison of the overall WOMAC score, patients were classified into four groups, as follows: > 75 points equals to a very satisfied patient (i.e. excellent WOMAC results), range from 56 to 75 points equals to a satisfied patient (i.e. good WOMAC results), range from 43 to 55 points signifies a dissatisfied patient (i.e. poor WOMAC results), < 43 points equals to a very dissatisfied patient and relates to a very poor WOMAC score. In our study, according to the above classification, the results were more than satisfactory. The majority of our patients were in the first WOMAC group, both for the CR and the KR prostheses; the percentage of the overall WOMAC score listed, respectively, for the CR 74.1, 21.6, 2.5 and 1.9% and for the KR 75, 20.6, 4.2 and 0%.

The most common complication in both groups was residual pain, which patients reported as mild, sometimes after overuse/ overload: 29.6% in the CR group, and 25% in the KR group (*p* = 0.57), which is the same percentage or less than reported by some authors in the literature. Parvizi et al. [3] described a high overall percentage of residual pain in younger patients with an average age of 56 ± 14.1 years, with 33% of patients reporting some degree of pain, 41% stiffness, 33% unpleasant crepitations during movement, 33% knee swelling, 38% problems getting in and out of the car, 31% had problems getting up and sitting on a chair, and 54% reported having difficulty walking up the stairs. Parvizi et al. [3] also pointed out in their study that even though patients are operated by experienced surgeons in large orthopaedic centres, several of them continue to suffer from residual pain or other difficulties leading to dissatisfaction with the outcome of the operation. In connection with their findings, it is recommended to thoroughly inform patients of the likelihood of some limitations and residual pain after TKA, and thus somehow adjust their expectations before surgery. Sekiva et al. [20] suggest arthroscopic debridement of scarred fibrotic tissue in each compartment of the knee as a possible source of pain after exclusion of an infection or aseptic TKA loosening. Following this procedure, Sekiva [20] reported that as much as 63% of patients were completely pain-free, 3% of patients reported significant improvement, 20% had half improvement, and 3% of patients had only minimal improvement, 11% of patients remained unchanged. We have not implemented this procedure at our clinic so far. A relatively small group of patients may be considered to have been included in their study (*n* = 30).

The second most common complication in our study was stiffness. Clement et al. [21] described long-term poor functional outcomes and low patient satisfaction if stiffness persists in the first year after surgery. In his study, 5% of patients reported the presence of stiffness after the first year, while in our study it was observed 6.7% in the CR and 9.7% in KR group (*p* = 0.72), which we considered satisfactory. Also, we positively evaluated the absence of more serious complications such as deep vein thrombosis, periprosthetic infection or aseptic TKA loosening in KR group during the reporting period. One revision TKA due to a periprosthetic infection occurred in the CR group (0.6%) which we considered to be highly acceptable.

At present, we have not seen a similar study in literature comparing these two designs, either prospectively or retrospectively. A similar comparison between a conventional endoprosthesis and new implant design was described by Ranawat et al. [22] in a prospective study. Authors compared clinical and radiological results at two years after surgery between a conventional PFC Sigma prosthesis and a new design of Attune implant from the same manufacturer (DePuy Synthes, USA). The main difference between these two endoprosthesis is in the trochlear groove shape. Ranawat et al. [22] assumed better functional score, better range of movement and even less complication with the new Attune endoprosthesis. In this study, he did not find a statistically significant difference in the range of motion, nor in the functional score (KSS 1 and KSS 2), nor among the patient satisfaction, evaluated with the VAS score. On the other hand, a significant lower percentage of anterior knee pain was observed in the Attune group in comparison of the Sigma group (12.5 vs. 25.8%, *p* = 0.02), as well as a lower percentage of patients who felt or heard peeling, crepitations from the knee endoprosthesis while moving (17.7 vs. 30.9%, *p* = 0.02). It is important to highlight that these two implants were posterior-stabilised knee endoprosthesis.

We realize that our study presented many limitations. Prospective randomised comparative studies with a larger group of patients need to be conducted, and long-term results should also be assessed. Since many factors influence the surgery outcomes, including patient expectations [9, 20, 23, 24], the patient's mental condition before surgery [23, 25, 26], as well as the presence of other comorbidities [19, 23, 27], is also important to record these attributes when evaluating the results. The impact of one-time bilateral knee replacement on functional outcomes in groups, which we did not analyse in our study is questionable [28–32]. Krivanek et al. [33] reported that there were no statistically significant differences in functional outcomes in the short-term follow-up between single bilateral and unilateral total knee replacement, although range of movement may be higher in the bilateral group. The influence of the learning curve may also be significant, as these were the first patients who underwent knee replacement using Physica KR in Slovakia and Czech Republic.

## Conclusions

Our study showed a statistically significant difference in functional results at three years with better KSS 1 scores, a tendency to higher values in the KSS 2 score, as well as a statistically significant overall improvement in active range of movement in favour of KR TKA over CR TKA with post-hoc power analysis at 83.8%. On the other hand, we found that there was no statistically significant difference between the two groups when comparing WOMAC score and the incidence of complications at short-term follow-up. More component sizes available, and thus more accurate adaptation to each patient specific anatomy, along with the results of our study, favour the KR prosthesis in primary TKA when retaining the posterior cruciate ligament. Further, we plan to evaluate larger groups of patients using new KR system and to focus on radiological and functional outcomes from mid- to long-term follow-up.
